# Comparison of Sexually Transmitted Infections and Adverse Perinatal Outcomes in Underserved Pregnant Patients Before vs During the COVID-19 Pandemic in Texas

**DOI:** 10.1001/jamanetworkopen.2022.0568

**Published:** 2022-02-15

**Authors:** Irene A. Stafford, Jennie O. Coselli, Danielle F. Wilson, Courtney Y. Wang, Baha M. Sibai

**Affiliations:** 1 Department of Obstetrics, Gynecology, and Reproductive Sciences, McGovern Medical School, The University of Texas Health Science Center at Houston, Houston, Texas

## Abstract

This cohort study assesses the rates of sexually transmitted infections (STI) and associated adverse perinatal outcomes among underserved pregnant patients during the COVID-19 pandemic in a public health system in Southeastern Texas.

## Introduction

Before the COVID-19 pandemic, the US experienced record high rates of many reportable sexually transmitted infections (STI).^1^ The association between the pandemic and STI rates among pregnant women remains understudied, especially for underserved populations of racial and ethnic minority groups who have disproportionately experienced COVID-19 infection.^2^ We sought to determine rates of STI and associated adverse perinatal outcomes among underserved pregnant patients before (July 2019 to February 2020) and during the pandemic (March 2020 to April 2021) in a public health system in Southeastern Texas.

## Methods

A retrospective cohort study was performed of pregnant patients between July 2019 and April 2021 within a public health system in Southeastern Texas. The University of Texas Health Science Center-McGovern Medical School institutional review board approved this study with waiver of informed consent granted because it involved materials that had been previously collected. Medical record review was performed for all patients who underwent routine STI screening during pregnancy and delivered during the study period. This study followed the Strengthening the Reporting of Observational Studies in Epidemiology (STROBE) reporting guideline for cohort studies.

Coprimary outcomes included a comparison of rates of individual and composite STI before the pandemic and during the pandemic along with a composite of STI-associated adverse maternal and neonatal outcomes. Rates of STI according to COVID-19 positive status were also calculated. Demographic, obstetric, and neonatal outcomes were recorded, including antenatal STI and COVID-19 infection (eMethods in the Supplement).

The maternal composite included hypertensive disorders, infectious morbidity, emergency postpartum visits, or death. The neonatal composite included neonatal intensive care admission, 5-minute Apgar score less than 7, sepsis, seizures, necrotizing enterocolitis, intraventricular hemorrhage, hydrops fetalis, or death. The χ^2^ test and multivariable regression were used for analysis. Models were adjusted for insurance, parity, prenatal care, body mass index, and group B *Streptococcus* status. Statistical significance was set at a 2-sided unpaired *P* < .05. Data were analyzed using STATA, version 15.0 (StataCorp).

## Results

The study population included 6413 patients (mean [SD] age, 29.2 [6.6] years), of which 87% (5565) had analyzable data. Of these, 2412 patients (43%) delivered before the pandemic and 3153 patients (57%) delivered during the pandemic. There were no significant differences in baseline demographic and obstetrical variables between groups. Seven percent (226 of 5565) tested positive for COVID-19 infection during pregnancy.

There was no increase in composite or individual STI over time, except for syphilis infection, which nearly doubled across periods (prepandemic, 0.9% [22 of 2412] vs postpandemic, 1.5% [48 of 3152]; *P* < .004) (adjusted relative risk, 1.73; 95% CI, 1.04-2.86) (Table 1). Syphylis infection tripled among individuals with COVID-19 positivity during the pandemic (COVID-19 negativity, 1.3% [39 of 2936] vs COVID-19 positivity, 4.2% [9 of 216]; *P* < .001) (adjusted relative risk, 2.82; 95% CI, 1.37-5.79) (Table 2).

**Table 1.** Rates of STI Before the COVID-19 Pandemic vs During the COVID-19 Pandemic

Variable	No./total No. (%)		*P* value	RR (95% CI)	
	Before pandemic (July 2019-February 2020	During pandemic (March 2020-April 2021		Unadjusted	Adjusted^a^
STI composite	105/2412 (4.4)	162/3118 (5.2)	.15	1.19 (0.94-1.52)	1.20 (0.94-1.54)
Chlamydia	36/2412 (1.5)	57/3115 (1.8)	.33	1.23 (0.81-1.85)	1.15 (0.74-1.79)
Gonorrhea	11/2412 (0.5)	16/3115 (0.5)	.76	1.13 (0.52-2.42)	NC
Syphilis	22/2412 (0.9)	48/3152 (1.5)	.04	1.67 (1.01-2.76)	1.73 (1.04-2.86)
HIV	8/2412 (0.3)	8/3152 (0.3)	.59	0.77 (0.29-2.04)	NC
Hepatitis B	34/2412 (1.4)	43/3152 (1.4)	.89	0.97 (0.62-1.51)	0.95 (0.61-1.48)

Abbreviations: NC, not calculable (due to infrequent or 0 events); RR, relative risk; STI, sexually transmitted infections.

^a^ Adjusted for insurance, parity, prenatal care, body mass index, and group B *Streptococcus* status.

**Table 2.** STI vs COVID-19 Infection During COVID-19 Pandemic (March 2020-April 2021)

Variable	No./total No. (%)		*P* value	RR (95% CI)	
	COVID-19 negativity	COVID-19 positivity		Unadjusted	Adjusted^a^
STI composite	144/2906 (5.0)	18/212 (8.5)	.03	1.71(1.07-2.74)	1.48 (0.93-2.35)
Chlamydia	51/2904 (1.8)	6/211 (2.8)	.26	1.62 (0.70-3.73)	1.18 (0.52-2.70)
Gonorrhea	14/2904 (0.5)	2/211 (0.9)	.30	1.97 (0.45-8.60)	NC
Syphilis	39/2936 (1.3)	9/216 (4.2)	.001	3.14 (1.54-6.39)	2.82 (1.37-5.79)
HIV	8/2936 (0.3)	0/216 (0.0)	>.99	NC	NC
Hepatitis B	41/2936 (1.4)	2/216 (0.9)	.77	0.66 (0.16-2.72)	NC

Abbreviations: NC, not calculable (due to infrequent or 0 events); RR, relative risk; STI, sexually transmitted illness.

^a^ Adjusted for insurance, parity, prenatal care, body mass index, and group B *Streptococcus* status.

Between the prepandemic-and during-pandemic periods, there were no differences in the risk of STI-associated composite maternal adverse outcomes (prepandemic, 11.4% [12 of 105] vs postpandemic, 17.9% [29 of 162]; *P* = .15) (adjusted relative risk, 1.27; 95% CI, 0.67-2.41). There were no differences in the risk of STI-associated composite neonatal adverse outcomes (prepandemic, 14.3% [15 of 105] vs postpandemic,14.5% [23 of 159]; *P* = .97) (adjusted risk ratio, 1.02; 95% CI, 0.54-1.93).

## Discussion

Results of this cohort study suggest that syphilis infection significantly increased among underserved pregnant patients delivering in Southeastern Texas during the COVID-19 pandemic, with rates as high as 4% for individuals with SARS-CoV-2 infection. These results are consistent with data presented by the Centers for Disease Control and Prevention indicating that STI disproportionately affects racial and ethnic minority groups who experience higher rates of infection with COVID-19 and health care disparities.^1,2,3,4,5^

Reallocation of federal and state STI prevention programs to serve in the COVID-19 response has led to disruptions to health care services, limiting access to public STI clinics. According to the National Coalition of STD Directors,^3^ 98% of sexually transmitted disease (STD) programs have added COVID-19 tracking efforts to their prioritized STD/HIV caseloads, substantially delaying STD surveillance response. This delay may have contributed to the increasing rates of syphilis.^1,2,3,4^

Although current guidelines^1^ stipulate that all pregnant women be screened for these STI during pregnancy, a limitation of this study is that it is unknown whether these patients were infected before becoming pregnant given the challenges associated with sexual health visits during the pandemic.^1,2,3,4,5^ This limitation is unlikely to explain these differences in rates of syphilis across these periods.

There has been a resurgence of STI, especially among medically underserved minority female populations who may have deferred sexual health visits, regardless of continued risk factors, such as partner violence, transactional sex, reduced access to known sexual health services, fear of discrimination, and stigmatization regarding STI.^4,5^ Women with asymptomatic STIs, such as syphilis, may be at highest risk, given the vague and overlapping symptoms suggestive of other conditions.

The results of this cohort study underscore the need for continual investment in public surveillance efforts for STI, especially for underserved pregnant patients who remain at highest risk for SARS-CoV-2 infection and associated maternal morbidity.

## Notes

Supplement. **eMethods.** Patient Identification and Screening
