# Supplementary material for: Comparison of Sexually Transmitted Infections and Adverse Perinatal Outcomes in Underserved Pregnant Patients Before vs During the COVID-19 Pandemic in Texas
Source: JAMA Netw Open. 2022 Feb 15;5(2):e220568. doi: 10.1001/jamanetworkopen.2022.0568 (PMC8848206; doi:10.1001/jamanetworkopen.2022.0568)
Supplement: Supplement. — eMethods. Patient Identification and Screening [file jamanetwopen-e220568-s001.pdf]

Supplemental Online Content

Stafford IA, Coselli JO, Wilson DF, Wang CY, Sibai BM. Comparison of sexually transmitted infections and adverse perinatal outcomes in underserved pregnant patients before vs during the COVID-19 pandemic in Texas. *JAMA Netw Open.* 2022;5(2):e220568. doi:10.1001/jamanetworkopen.2022.0568

eMethods. Patient Identification and Screening

This supplemental material has been provided by the authors to give readers additional information about their work.

© 2022 Stafford IA et al. *JAMA Network Open*.

eMethods. Patient Identification and Screening

Patients were identified using ICD-10 codes related to STI screened for during pregnancy (N. gonorrhea, C. trachomatis, Human Immunodeficiency virus, syphilis, hepatitis B virus) Hepatitis C screening was not implemented until 2020 therefore it was not queried for this project. HSV was not queried due to inability to discern serologic evidence of infection versus history of infection using ICD-10 codes. In addition, ICD-10 codes were used to identify maternal and neonatal comorbidities of interest. Because ICD10 codes are reliant on healthcare providers or the billing department selecting a diagnosis code, the ICD-10 code selection was expanded to include diagnosis such as diabetes, gestational diabetes, hypertensive disorders, including mild and severe preeclampsia and eclampsia and chronic hypertension. Also, ICD-10 was used to identify prior preterm birth, prior cesarean delivery, thyroid disease and Sars-Co-V2 positive status. Procedural codes and demographic variables that were available via EMR search were used with IRB approval. These were used to also capture mode of delivery and maternal/neonatal intensive care admission and ICD-10 codes were used to query neonatal outcomes as contained in chart below. Chart review was performed on 2021 charts to confirm validity and accuracy of coding and ICD-10 codes with patient diagnoses by PI. A power analysis was not performed a priori as the study population for this observational study was restricted by a fixed time period (pre and during the pandemic)

Item to Display	Description
name	Query
mrn	Query
Date of birth	Query
Age	Query
Race	Query
Ethnicity	Query
Insurance	Query
Preferred language (if available)	Query
Marital status	Query
Parity	Query
Number of total prenatal care visits with Harris Health	Query
Insufficient prenatal care*	O09.3
Acute respiratory infection (COVID-19)	U07.1, J12.82

© 2022 Stafford IA et al. *JAMA Network Open*.

Chronic hypertension	O10, O10.0, O10.2-.319, O10.4, O10.41 -.43, O10.9, O10.02, O10.93
Gestational hypertension	O13.1 - .9
Mild preeclampsia preeclampsia	O13, O14.0, O14.1, O14.2, O14.9, (mild O14.03)
Gestational diabetes	O24.41 - .44
Type I diabetes	O24.011 - .019
Type II Diabetes	024.1<br><br>O24, O24.0, O24.01 -.03, O24.11 -.13, O24.3, O24.31 - .33, O24.8, O24.81 -. 83, O24.9, O24.91-.93
Thyroid disease	E07.9<br><br>O99.280
Prior preterm birth	Z87.51
Prior cesarean delivery	O34.21
STI list (Human immunodeficiency virus, Chlamydia trachomatis, Neisseria Gonorrhoeae, syphilis)	O98.1, O98.11 -.13, O98.2, O98.22, O98.23, O98.3, O98.31-.33, O98.7, O98.71 - .73, O98.4, O98.41, O98.43, O98.8, O98.81 -.83, O98.9, O98.91 -.93
Admission diagnosis labor or active labor	O75.02<br>075.82
Admission Dx preterm labor	O60, O60.0, O60.1 -.3
Preterm premature rupture of membranes	O42.00, O42.01, O42.011 - ..013, O42.0139, O42.10, O42.11, O42.111 -.113, O42.91, O42.911, O42.912, O42.913. O42.919
Gestational age at time of admission	Query
Insufficient prenatal care*	O09.3
Mode of delivery (cesarean or vaginal) *	O80, O80.0, O80.1, O80.8, O80.9, O81, O81.0, O81.1 -.4, O81.5, O82, O82.0, O82.2,

© 2022 Stafford IA et al. *JAMA Network Open*.

	O82.8, O82.9, O83, O83.0, O83.1 -.4, O83.8, O83.9, O84.0, O84.1, O84.2, O84.8, O84.9
Intensive care unit admission (linked to admission for delivery)	Query
Sepsis	O85, O75.3
Wound infection	O86.0
Maternal death	O95, O96, O96.0, O96.1, O96.9, O97, O97.0, O97.1, O97.9, P01.6
Post partum hemorrhage	O72.1, O72.2
Blood transfusion	Query
Chorioamnionitis	O41.1, O41.10, O41.121 - .123, O41.129
Endomyometritis	O85, O86.1, O86.11, O86.12, O86.19, N80.9
Group B streptococcus	O99.82, O99.820, O99.824, O99.825
Length of hospital stay - mother	Query
Seen in ER for up to 6 weeks postpartum? (Y/N)	Query
Postpartum readmission up to 6 weeks (Y/N)	Query
Newborn weight	Query
Newborn sex	Query
Neonatal Apgar scores (1 min, 5 min)	Query
Neonatal intensive care unit admission	Query
Neonatal sepsis	P36, P36.0, P36.1, P36.10, P36.19, P36.2, P36.30, P36.39, P36.4, P36.5, P36.8, P36.9

© 2022 Stafford IA et al. *JAMA Network Open*.

hydrops	P56.0, P56.90-.99, P83.2, O36.2, O36.20X0, O36.20X1 -5, O36.20X9, O36.21X0, O36.21X1 -X5, O36.21X9, O36.22, O36.22X1 -X4, O36.22X9, O36.23. O36.23X0-9. O36.23X1 -X5
Necrotizing enterocolitis	P77, P77.1 -.3, P77.9
Intraventricular hemorrhage	P10.2, P52, P52.0 -, P52.2,2 P52.3
Respiratory distress syndrome	P22, P22.0, P22.1, P22.8, P22.9, ZO5.3
seizures	P90
Neonatal death	Query
Large for gestational age	P08.1
Small for gestational age	P05.10
Intrauterine fetal demise	O36. 4

© 2022 Stafford IA et al. *JAMA Network Open*.
